# Results of a sector-wide quality improvement initiative for substance-abuse care: an uncontrolled before-after study in Catalonia, Spain

**DOI:** 10.1186/1747-597X-5-26

**Published:** 2010-10-23

**Authors:** Pilar Hilarion, Oliver Groene, Joan Colom, Rosa M Lopez, Rosa Suñol

**Affiliations:** 1 Avedis Donabedian University Institute, Autonomous University of Barcelona (UAB), C/Provenza 293, Pral, 08037 Barcelona, SPAIN; 2 CIBER Epidemiology and Public Health, Doctor Aiguader, 88 1a Planta, Barcelona, SPAIN; 3 Program on Substance Abuse. Department of Health. Government of Catalonia, SPAIN

## Abstract

**Background:**

The Health Department of the Regional Government of Catalonia, Spain, issued a quality plan for substance abuse centers. The objective of this paper is to evaluate the impact of a multidimensional quality improvement initiative in the field of substance abuse care and to discuss potentials and limitations for further quality improvement.

**Methods:**

The study uses an uncontrolled, sector-wide pre-post design. All centers providing services for persons with substance abuse issues in the Autonomous Community of Catalonia participated in this assessment. Measures of compliance were developed based on indicators reported in the literature and by broad stakeholder involvement. We compared pre-post differences in dimension-specific and overall compliance-scores using one-way ANOVA for repeated measures and the Friedman statistic. We described the spread of the data using the inter-quartile range and the Fligner-Killen statistic. Finally, we adjusted compliance scores for location and size using linear and logistic regression models.

**Results:**

We performed a baseline and follow up assessment in 22 centers for substance abuse care and observed substantial and statistically significant improvements for overall compliance (pre: 60.9%; post: 79.1%) and for compliance in the dimensions 'care pathway' (pre: 66.5%; post: 83.5%) and 'organization and management' (pre: 50.5%; post: 77.2%). We observed improvements in the dimension 'environment and infrastructure' (pre: 81.8%; post: 95.5%) and in the dimension 'relations and user rights' (pre: 66.5%; post: 72.5%); however, these were not statistically significant. The regression analysis suggests that improvements in compliance are positively influenced by being located in the Barcelona region in case of the dimension 'relations and user rights'.

**Conclusion:**

The positive results of this quality improvement initiative are possibly associated with the successful involvement of stakeholders, the consciously constructed feedback reports on individual and sector-wide performance and the support of evidence-based guidance wherever possible. Further research should address how contextual issues shape the uptake and effectiveness of quality improvement actions and how such quality improvements can be sustained.

## Background

Over the last decade a multitude of performance assessment and quality improvement activities have targeted the health care sector. In addition to regional and national initiatives, efforts led by the World Health Organization or the Organization for Economic Development and Cooperation aim at assessing and evaluating health care and health systems in an international comparative perspective [1-3]. Indeed, industrial approaches to quality improvement and performance assessment have been applied to health care since the 1960s, spearheaded by the works of Avedis Donabedian [4,5]. While most attention has initially focused on the hospital setting and primary care, more and more attention is being been paid on other health and social care facilities in general, and mental health [6-8] and substance-abuse centers in particular [9-12].

Relevant initiatives to improve the quality in this sector are for example the treatment protocols and technical assistance publications (including models of good practice) that are being prepared by the US Center for Substance Addiction Treatment [13]; the Accreditation Manual for Behavioral Care issued by the Joint Commission [14], or the epidemiological studies and reports that are linked to evaluation and improvement efforts published by the European Monitoring Center for Drugs and Drug Addiction [15]. Moreover, traditional quality management models, such as the European Foundation for Quality Management (EFQM) model, have been applied to substance abuse centers [16]. In Spain, too, various Autonomous Communities have launched activities to improve the quality of substance-abuse centers, for example the evaluation based on the Common Assessment Framework carried out by the Government of Cantabria, the assessment of compliance of substance abuse centers with a set of standards conducted by the Institute for Addiction Research in Madrid and the quality plan and external evaluation system deployed by the Catalonian Government to improve substance abuse services [17]. We described the conceptual basis of the latter system in a previous article [18].

In the following, we provide a more in-depth application of the evaluation framework widely used in health care to centers that provide ambulatory substance abuse services for people with drug dependencies. In order to address the quality issues of concern in these centers (adherence to treatment, continuity of care) and the multiple factors causing them, quality improvement efforts need to adapt a multi-professional approach that takes into consideration social, clinical, psychological factors as well as the user's views and satisfaction. The objective of this paper is thus to evaluate the impact of a multidimensional quality improvement initiative in the field of substance abuse care in the Autonomous Region of Catalonia, Spain, and to discuss potentials and limitations for further quality improvement.

## Methods

### Setting

This study was conducted in the context of the quality plan issued by the Health Department of the Autonomous Government of Catalonia. The plan states the need for quality improvement efforts directed at centers for substance-abuse service and follow up (CAS), with particular attention on existence of assessments of quality as perceived by the users of the centers. CAS provide services by multi-professional teams including physicians, psychologists, social works and nurses that review on a case by case basis the needs and treatment forms for each patient and assess further health and social care needs, such as the use of therapeutic communities or hospitalization in an detoxication unit. These centers can offer different treatment options such as methadone treatment or treatment with narcotic antagonists and a like. In addition, some CAS are linked to small peripheral centers that facilitate for example medication dispensing. Performance of these small units is not included in the analysis presented here.

### Measures and data sources

Considering that developing performance measures can be very resource demanding, we developed the measures for external evaluation based on reviews of the literature, consensus methods including subject experts and involving a broad range of stakeholders. The key premise in this process is to address issues that are of concern of practicing health and social care professionals, to include the latest scientific evidence and expert knowledge in the definition of indicators and to ensure practicality and timeliness of the work. The measurement development would first draw on previously reported and validated survey measures, data collection instruments and indicators. Multi-disciplinary stakeholder groups then meet five to seven times to discuss how the measures reflect the main problems in the sector, identify objectives that can be met within three years, consent on the key measurement area and review, prioritize and technically define the measures. The development and psychometric testing of new indicators that require complex surveys or clinical scores is not the intention and is beyond the scope of our work. Indicators are defined using a standardized template. For each indicator, standards are set based on a review of the literature and consensus in the group. Standards are set to encourage improvement as considered to be achievable by stakeholders within the next 2 or 3 years, after which they will be measured again and a new set is developed. Indicators for which a majority of centers are already performing very well will be discarded. The process of measure development is described in more detail elsewhere [18,19].

Data sources used for the external evaluation depended on the criteria and dimension of care assessed. This includes revisions of clinical records for care processes and follow-up indicators, direct observation for structural indicators, harm reduction program and assessment of systems for user evaluation, and revision of specific documents such as program descriptions, protocols and minutes for the remaining indicators. For standardization and quality assurance purposes, all data are recorded in a specifically developed program in MS ACCESS.

For indicators based on user records we took a random sample of 100 records based on the user lists provided by the centers. All patients are included at the sampling stage. In the instance of small centers not reaching this number we assessed all cases. Data extraction is based on an algorithm taking into account the type of indicator, non-applicability of measurable elements and level of compliance. These measures are then aggregated at indicator level (an indicator is typically based on around four measurable elements) and in a subsequent step at dimension level. The original data format specifies for each center, indicator and assessment period (pre-post) whether the predetermined standards were met. Based on the indicator-specific dichotomous compliance scores we calculated center-level compliance scores for each performance dimension. The resulting scores can be expressed as follows:

• **Overall compliance**: for each centre, the total number of indicators that are in compliance divided by the total number of indicators evaluated (where "Compliant/NotCompliant" is ***a dichotomous variable ***(**1 **= *compliant*, **0 **= *not compliant*) and *m *is the number of indicators evaluated.

$$OverallCompliance=\frac{\sum_{i=1}^{m}Compliant/NotCompliant}{m}\times 100,$$

• **For each of the 4 dimensions**: for each centre, the total number of indicators related to the respective dimension divided by the total number of indicators evaluated for the same dimension (where "Compliant/NotCompliant" is ***a dichotomous variable ***(**1 **= *compliant*, **0 **= *not compliant*) and "*m *Indicadors Evaluated in the dimension" is the number of indicators evaluated):

$$DimensionA=\frac{\sum_{i=1}^{m}IndicatorEvaluatedDimension_{Compliant/NotCompliant}}{mIndicatorsEvaluatedDimension}\times 100$$

These resulting scores are continuous (except for the dimension 'environment and infrastructure' as this is composed of only one indicator) and allow for the comparison of pre-post compliance scores. While the aggregate measures account for applicability of the item assessed and for missing data, we did not weight individual indicators. For example, the correct registration of drug prescriptions is not assigned more weight than, say, providing vaccination against Hepatitis B to users at risk. The rationale for this is that given the different views in the multi-disciplinary measure development stakeholder group (involving users) we could not consent on criteria that would justify weighting of indicators.

### External evaluation and quality improvement

Centers were informed before the visit about the evaluation schedule and the documents required during the evaluation. Baseline evaluation was carried out in 2001 and follow up evaluation was performed in 2004. Between the two evaluations, centers received specific performance reports including targeted recommendations for quality improvement. At the same time a sector-wide report was published including the baseline assessment (without identifying individual providers) and recommendations for sector-wide improvements. Evaluators received a 40 hour training for the assessment exercise including a presentation of the indicators, data sources, the assessment instrument and overall methodology. A pilot test was carried out to test the assessment material and the reliability of the assessment by different evaluators. The assessment of inter-rater reliability itself is outsourced to another scientific institute (the Ibero-American Cochrane Collaboration, Barcelona) to limit any biases results from personal acquaintance with the evaluators.

Subsequent to the evaluation, and in order to facilitate quality improvement initiatives in the participating centers, each center received an individualized quality improvement report. This report indicates the results of the assessment, offers concrete proposals for quality improvement and provides anonymized comparative data on other center's performance. In addition, a sector wide report is prepared to address quality issues beyond individual center's research and is discussed at sector level, involving health policy and other stakeholders. Based on the later report, quality improvement actions are consented at sector level.

### Data analysis

We computed indicator-, dimension-specific and overall compliance scores based on the calculation of compliance with measurable elements. Using these scores we present overall and dimension-specific results for baseline and follow up evaluation with descriptive statistics of tendency and variation. We performed one-way analysis of repeated measures (ANOVA) and the Friedman statistic to assess the level of statistical significance for differences between baseline and follow-up evaluation. To test whether sector wide changes in performance are associated with reduced spread in individual center's performance we assessed changes in inter-quartile range and the Fligner-Killeen statistic for the performance dimensions between baseline and follow-up assessment. Finally, in order to control for the effect of the structural center-characteristics 'location' and 'size', we entered these variables into multiple linear and logistic regression models. Data analysis was performed using SPSS 17 and the R statistical software package (version 2.10.1).

## Results

We assessed and followed up all 22 Centers for Attention and Follow Up participating in the quality plan and providing services to persons addicted to drugs and other substances in the Autonomous Community of Catalonia, Spain (reflecting the whole sector). In Table 1, we provide an overview on the distribution of centers by geographic territory as well as descriptive statistics on the number and characteristics of their users (Table 1).

**Table 1:** Participating centers: distribution and users

Characteristic	
Total number of centers (% of the sector)*	22 (100%)

Centers by geographic territory (%)	
*Barcelona*	16 (72.7%)
*Girona*	1 (4.5%)
*Lleida*	2 (9.1%)
*Tarragona/Delta de l'Ebro*	3 (13.6%)

Number of active users	
*Total users*	13701
*Mean/median users per center*	622.8; 540.0
*Standard deviation*	399.4
	
*Users on methadone*	4006
*Mean/median users per center*	182.1; 160.0
*Standard deviation*	127.4

The majority of centers for attention and follow up, corresponding to the highest concentration of need and demand, are based in the Barcelona region. The standard deviations for the reported means on active users reflect that the size of the centers in terms of users (but also in terms of professionals and infrastructures) differs widely. It took an average one-person day to perform the evaluation in each center.

All Centers for Attention and Follow Up were evaluated twice using consensus indicators developed jointly with the stakeholders. These indicators were designed to be applicable to all centers. Table 2 gives an overview on the indicators, their relation to the multidimensional evaluation framework and their distribution across dimensions, themes, indicators, and measurable elements. Overall, the evaluation framework includes 4 dimensions, 20 themes, 35 indicators and 169 measurable elements (Table 2).

**Table 2:** Evaluation of individual center's performance: domains, themes, indicator and measurable element

Domain	Theme	Indicator	N (cases revised)*	Standard	Compliance		
					
					At baseline	At follow up	Change
**Care pathway**	Process of admittance	1. Ensure follow up visits after user contacts and hospitalizations.	2476	75%	100.0%	100.0%	**0.0%**
	
	Process of care	2. Offer an individualized treatment plan in appropriate timeframe.	1312	90%	100.0%	95.5%	**-4.5%**
		
		3. Offer vaccination against hepatitis B to all users at risk.	757	90%	40.9%	63.6%	**55.5%**
		
		4. Provide access to counseling services to HIV positive users.	703	85%	4.5%	57.1%	**1168.9%**
		
		5. Provide a list of treatments and services, including a description of the nature of the intervention.	184	100%	45.5%	86.4%	**89.9%**
	
	Pharmacological treatment	6. Register patients drug prescriptions properly.	1223	100%	72.7%	90.9%	**25.0%**
	
	Follow up care	7. Follow up users in the methadone maintenance programme at least every 6 months.	1119	90%	90.9%	77.3%	**-15.0%**
		
		8. Support the user in adhering to the care pathway.	14256	85%	65.0%	86.4%	**32.9%**
		
		9. Actively follow up patients that do not attend the dispensing of methadone.	542	90%	50.0%	50.0%	**0.0%**
	
	Prevention	10. Promote and participate in prevention activities (on own initiative, in coordination with community agencies or by indication of agencies).	64	100%	95.5%	95.5%	**0.0%**
	
	Harm reduction program	11. Have standardized guidelines for the prevention of risk behaviors associated with substance use and sexual behavior.	152	100%	63.6%	90.9%	**42.9%**
		
		12. Provide injection equipment (syringes) to intravenous drug users.	52	100%	59.1%	100.0%	**69.2%**
		
		13. Have programs aimed at (potential) users not yet in contact with the center.	53	100%	86.4%	90.9%	**5.2%**

**Relations and user rights**	Confidentiality	14. Ensure confidentiality of all user-related data.	83	100%	40.9%	72.7%	**77.7%**
	
	Information	15. Ensure that users have all the necessary information to take an informed decision regarding all health-related actions.	1150	100%	4.5%	22.7%	**404.4%**
	
	User satisfaction	16. Demonstrate a system of dealing with complaints (ensuring feedback within two weeks)..	59	80%	80.0%	76.9%	**-3.9%**
		
		17. Assess the satisfaction of users with the services received.	100	100%	36.4%	36.4%	**0.0%**

	Family involvement	18. Have an action plan with families, encompassing monitoring and periodic contacts, every six months.	683	80%	90.9%	86.4%	**-4.9%**
	
	Community involvement	19. Promote the reintegration of the user in the community.	528	80%	100.0%	95.5%	**-4.5%**
		
		20. Engage in efforts to improve community acceptance.	57	100%	100.0%	95.5%	**-4.5%**
		
		21. Conduct activities to improve social acceptance of care in collaboration with associations, councils, regional councils, etc.	56	90%	86.4%	95.5%	**10.5%**

**Environment and infrastructure**	Appropriateness of the facilities	22. Designate a space reserved for the intake and dispensing of methadone.	53	100%	81.8%	95.5%	**16.7%**

	Organization and waiting times	23. Provide written information to the population at risk about the services, including information on treatment, hours, place of care.	124	100%	40.9%	95.5%	**133.5%**
		
		24. Anticipate the provision of services to patients outside opening hours.	61	100%	63.6%	81.8%	**28.6%**
		
		25. Initiate diagnosis and therapy in a period no longer than 2 weeks of initial visit.	1325	80%	88.2%	76.2%	**-13.6%**
	
	Documentation systems and registries	26. Document the clinical history for all users actively attended in center.	1266	100%	59.1%	86.4%	**46.2%**
		
		27. Make accessible the clinical documentation generated during the visits to all members of the multi-professional team.	1321	95%	95.5%	100.0%	**4.7%**
	
**Organization and management**	Multi-professionalcare	28. Assess all patients in the care at least once by the professionals who comprise the multi-professional team.	1197	75%	31.8%	63.6%	**100.0%**
	
	Protocols	29. Demonstrate protocols for the triage of users with organic pathologies.	159	100%	36.4%	90.9%	**149.7%**
		
		30. Demonstrate protocols for the triage of users with psychopathologies.	81	100%	45.5%	72.7%	**59.8%**
		
		31. Demonstrate protocols for the triage of pregnant woman users.	101	100%	40.9%	90.9%	**122.2%**
	
	Continuing education	32. Professional should participate in continuing education activities.	375	80%	45.5%	63.6%	**39.8%**
	
	Professionals' opinion	33. Carry out regular surveys on the opinion of professionals.	57	100%	18.2%	40.9%	**124.7%**
	
	Coordination with other levels of care	34. Establish stable relationships and coordination with affiliated social services and legal agencies.	170	100%	45.5%	68.2%	**49.9%**
		
		35. Coordinate work plan with the health care area administration, mental health centers and referral hospitals.	219	100%	54.5%	72.7%	**33.4%**

* the differences in the denominators between indicators is due to the level at which the indicators is selected: records (depending on type of user) at user level or organizational policies at center level.

The number of measurable elements for each indicator was determined based on the indications from stakeholders regarding key aspects to be assessed. The median number of measurable elements per indicator is 4, but some indicators are assessed by only one measurable element while others are assessed by up to 16. Evaluators assessing the indicators are specifically trained in data collection and use standardized algorithms and data entry sheets for data collection.

The number of cases that forms the basis of the calculation of the indicators differs substantially as some indicators are based on the random review of a sample 100 user records, others are based on a full review of user records (in case the number of users for this indicator is <100 at center level) and again others are based on assessment at center level, for example, the external evaluators' assessment of the availability of certain organizational procedures.

For some indicators we observed negative changes in compliance ratings (indicators 2, 7, 16, 17, 19, 20, 25). In case of indicators 2 and 25, these negative changes can be explained by a substantial increase in demand that coincided with the evaluation period and centers were not prepared for this increase. For indicators 16, 17, 19 and 20 other institutions or third parties were established during the evaluation period and took over some responsibilities that were initially held at the level of the center (such as the complaint handling system). Finally, indicators 7 on the follow up of users proved to be very difficult to achieve due to the reluctance of those users already on methadone to establish contact with the CAS.

After the baseline evaluation using these indicators, a bundle of quality improvement actions were initiated both at the level of the sector and at the level of individual centers. Key to these quality improvement actions were the performance reports provided to each center which depict the center's performance in relation to the sectors performance. Thus, each center could identify targeted quality improvement actions in areas where the center's performance was below standard. In addition to these center-specific performance reports, a sector-wide performance report was published focusing on quality improvement actions that require consorted actions or changes in the normative framework. The following table gives an example of a performance report for one of the indicators (Table 3).

**Table 3:** Example of quality improvement report for one indicator

Criterion number	34
**Domain**	Organization and management

**Theme**	Coordination with other levels of care

**Indicator**	Establish stable relationships and coordination with affiliated social services and agencies competent in legal matters pertaining to the Department of Justice.

**Rationale**	The range of legal and social problems associated with drug use requires a suitable level co-operation and coordination with the network of social services and agencies in legal matters in the Department of Justice.
**Measurable elements**	This will be assessed in terms of:
	- stable mechanisms for interaction and coordination
	- an established work plan containing a schedule of meetings
	- communication channels
	- a registry system
	- specific protocols.

**Standard**	**100,0% (all measurable elements should be in full compliance with)**

**Sector performance (2001)**	**68.2% (the average compliance with the criterion in the overall center)**

**Your performance (2001)**	**47.3% (the compliance with the criterion in your center)**

**Results**	The average performance of this indicator in the sector (68.2%) is below the established standard. Of all centers in the sector, none was completely non-compliant and 15 of the 22 centers were fully compliant.
**Targets for quality improvement action**	Compliance with measurable elements was as follows:
	- Evidence of some coordination with other levels of care: 93,5%
	- Calendar of Meetings: 29,5%
	- Stable communication channels: 15,9%
	- Registration system: 13,6%
	- Consensus protocols of action: 40,9%
	- Referral protocols and tracking: 43,2%
	The main reason for non-performance of this indicator has been the lack of systematic work with the department of justice followed by lack of collaboration with other social services. Moreover, compliance with protocols for intervention and monitoring of cases is low. The areas indicated above should be the target of further improvement work.

The performance report provides information on comparative benchmarking for each indicator and pinpoints at measurable elements that were evaluated particularly low. Target audience of the report is the center's management and involved professionals. It should be noted that the performance report sent to each center includes such a detailed assessment for each of the 35 indicators, accompanied by graphical illustrations of relative performance.

After the publication of the performance reports, local quality improvement actions were established, implemented and evaluated in the follow-up evaluation. For example, centers participated in the public presentation of the results and in the elaboration of the document on good practices. The good-practice document provides for all indicators that were evaluated as below target performance detailed rationales, improvement steps and reference documents in order to facilitate the use of this information for quality improvement activities. Specific quality improvement actions initiated on the basis of the good-practice document may for example be the administration of a user satisfaction survey, the implementation of a new protocol or the introduction of an organizational policy towards collaboration with other providers of substance abuse services. Based on individual performance reports, some centers also requested specific advice from the research coordination team on how to improve the quality of services.

**Figure 1:**
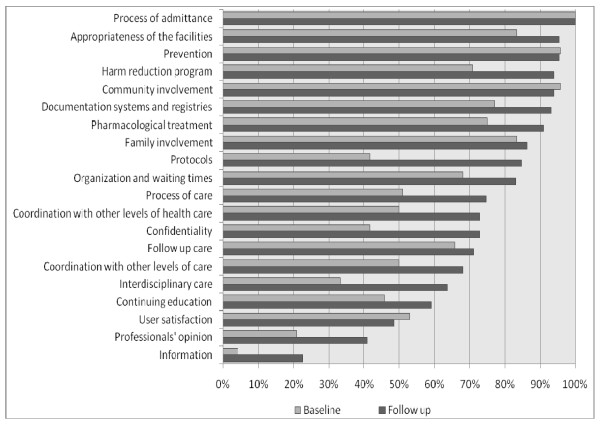
**Compliance at theme-level at baseline and follow up evaluation**.

Major improvements between baseline and follow up evaluation can be observed for most of the 20 themes evaluated (Figure 1, Results at the level of individual indicators are reported in Table 2). Exceptions can be observed in the four areas: adequate space, prevention, community relations and overall satisfaction. In the remaining 16 areas, major improvements were made at sector level.

In order to assess statistical significance for the differences observed, we carried out comparisons between baseline and follow up evaluation and performed a one-way ANOVA analysis for repeated measures and the Friedman statistic to assess the improvements in overall and dimension-specific performance (Table 4).

**Table 4:** Global results - baseline and follow up assessment

Domain	Mean compliance at baseline assessment	Mean compliance at follow up evaluation	Improvement in compliance (%; 95% confidence interval)	P-value for difference in compliance*	Statistic
*Overall compliance*^1^	60.9%	79.1%	29.9% (22.4%; 37.3%)	< 0.001	F_1,12 _= 47.40

*By dimension*					

Care pathway^2^	66.5%	83.5%	25.6% (15.0%; 36.1%)	0.002	Chi^2^_1_= 9.8

Relations and user rights^1^	66.5%	72.5%	9.0% (-5.9%; 23.9%)	0.124	F_1,21_= 2.57

Organization and management^2^	50.5%	77.2%	52.9% (37.8%; 67.9%)	< 0.001	Chi^2^_1_= 11.64

Environment and infrastructure^3^	81.8%	95.5%	13.63% (-0.7%; 28.0%)^4^	0.16 (0.002)^5^	T Wald = -2.97

^1^ANOVA ONE-WAY for repeated measures

^2 ^Friedman Test

^3 ^Logistic regression with dependent variable 'improvement in compliance'

^4 ^Hospitals that improved: % (95% CI)

^5 ^Odds-ratio (hospitals improved/hospitals did not improve: the probability to improve is significantly lower than 1 (0.136) at p-value 0.002.

Global improvement in mean compliance between baseline assessment and follow up evaluation is substantial (29.9%; 95% Confidence Interval (CI): 22.4-37.3; ANOVA, F_1, 21 _= 47.4). Since we were interested not only in the improvements of individual centres but also in the improvement of the sector as a whole, we assessed whether improvements observed are also accompanied by diminished variance between centers. The assessments using the inter-quartile range (IQR) yielded reductions for the dimensions 'overall compliance' (IQRpre: 0.179; IQRpost: 0.142) and 'care pathway' (IQRpre: 0.18, IQRpost: 0.14), but not for the dimensions 'relations and user rights' (IQRpre: 0.18, IQRpost: 0.30) and 'organization and management' (IQRpre: 0.16, IQRpost: 0.24). Given the dichotomous nature of the indicator for the dimension environment and infrastructure, we could not compute this statistic for this dimension.

In order to formally analyze the reduction in variance between pre-post assessments, we performed the Fligner-Killen test for changes in overall and dimension-specific compliance which yielded the following p-values: 'overall compliance' = 0.418 (Fligner-Killeen Test = 0.67; df = 1), 'care pathway' = 0.043 (Fligner-Killeen Test = 4.10; df = 1), 'relations and users rights' = 0.165 (Fligner-Killeen Test = 1.93; df = 1) and 'organization and management' = 0.963 (Fligner-Killeen Test = 0.002; df = 1). According the results of the Fligner-Killen test, the reduction in variance between pre and post assessment is only significant for the dimension 'care pathway'.

In terms of dimension-specific results, improvements ranging from 9.0% to 52.9% can be observed. Improvements were statistically significant in the dimensions 'care pathway' (pre: 66.5%, post: 83.5%; 95% CI for improvement in compliance: 15.0-36.1, Chi^2^_1 _= 9.8) and 'organization and management' (pre: 50.5%, post: 77.2%; 95% CI for improvement in compliance: 37.8-67.9, Chi^2^_1 _= 11.64). We observed improvements for the dimension environment and infrastructure' (pre: 81.8%; post: 95.5%) and the dimension 'relations and user rights' (pre: 66.5%; post: 72.5%), however, these improvements were not statistically significant.

In a final step, and in order to assess whether improvements in compliance are influenced by the location of the center or its size, we entered the following variables into a regression model: change in compliance between baseline and follow up evaluation, location and size. To this end we created dummy variables for location of the center (Barcelona vs. elsewhere) and for its size (small, medium, large; based on the distribution of the mean number of users per centers in terciles). Table 5 reports the improvement in overall and dimension-specific compliance by location and size of the center using multiple linear and logistic regression models. According to the model for 'overall compliance' (R^2 ^= 0.19, F_3, 18 _= 1.42), smaller centers reach higher improvements (B-value for 'big' compared to 'small' = -0.39) and centers in Barcelona improve more than others (B-value for 'Barcelona' = 0.65), however, these factors are not significant in the model (p-value = 0.27). Thus, it seems that the improvements presented in Table 4 are not confounded by these factors and centers appear to improve on the whole, irrespective of location and size.

**Table 5:** Improvement by location and size for overall and dimension-specific compliance

Domain	B	SE B	β (p-value)	Notes	Goodness of fit
**Overall Compliance**^1^	-2.19	0.33	(< 0.001)	R^2 ^= 0.19	F_3,18 _= 1.42 p-value = 0.27
- Constant^*2*^					
- Size	---	---	---		
*Small*	-0.33	0.38	-0.21 (0.393)		
*Medium*	-0.39	0.37	-0.25 (0.314)		
*Big*					
- Location	---	---	---		
*Elsewhere*	0.65	0.35	0.40 (0.079)		
*Barcelona*					

**Care pathway**^1^					F_3,18 _= 0.27 p-value = 0.84
- Constant	0.13	0.09	(0.195)	R^2 ^= 0.04	
- Size					
*Small*	---	---	---		
*Medium*	0.01	0.11	0.02 (0.934)		
*Big*	-0.04	0.11	-0.10 (0.692)		
- Location					
*Elsewhere*	---	---	---		
*Barcelona*	0.07	0.10	0.17 (0.489)		

**Relations and user right**^1^					F_3,18 _= 3.80 p-value = 0.03
- Constant	-0.12	0.06	(0.070)		
- Size				R^2 ^= 0.39	
*Small*	---	---	---		
*Medium*	0.07	0.07	0.23 (0.289)		
*Big*	0.04	0.07	0.12 (0.585)		
- Location					
*Elsewhere*	---	---	---		
*Barcelona*	0.19	0.06	0.54 (0.009)		

**Organization and management**^1^					F_3,18 _= 1.76 p-value = 0.19
- Constant	0.30	0.10	(0.011)	R^2 ^= 0.23	
- Size					
*Small*	---	---	---		
*Medium*	-0.24	0.12	-0.73 (0.055)		
*Big*	-0.17	0.12	-0.50 (0.168)		
- Location					
*Elsewhere*	---	---	---		
*Barcelona*	0.14	0.11	0.41 (0.211)		

**Environment and infrastructure**^3^					Residual Deviance on 18 degrees of freedom = 14.71 p-value = 0.68
- Constant	-0.96	1.19	0.38 (-4.00;1.21)^4^	0.421^5^	
- Size					
*Small*					
*Medium*	-0.69	1.40	0.52 (-3.92;2.08)^4^	0.648^5^	
*Big*	-18.45	4060.75	~0 (inf;inf)	0.996^5^	
- Location					
*Elsewere*	---	---	---	---	
*Barcelona*	-0.23	1.45	0.79 (-3.04;3.12)^4^	0.874^5^	

^1 ^Multiple linear regression

^2 ^Box-cox transformation (log)

^3^Multiple logistic regression

^4 ^Odds-ratio (95% CI)

^5 ^p-value

In the linear regression models for the dimensions 'care pathway' and 'organization and management' the factors location and size are not significant. However, the factor 'location = Barcelona' is significant in the model for the dimension 'relations and user rights' (p = 0.03, F_3, 18 _= 3.80, R^2 ^= 0.39). For the dimension 'environment and infrastructure' we fitted a logistic regression model, given the dichotomous nature of this dimension based on only one indicator. The odds-ratios and corresponding 95% confidence intervals do not suggest an influence of size and location on probability to improve.

## Discussion

We assessed and facilitated quality improvement activities in 22 Centers for Attention and Follow Up in the Autonomous Community of Catalonia, Spain. Using consensus indicators developed with the involvement of key stakeholders we carried out a baseline assessment, prepared targeted performance reports and performed a follow-up assessment. Overall, we observed substantial and statistically significant improvements in the whole sector. The results of the regression analysis in general do not suggest that centers in Barcelona and those being larger differ substantially in their compliance scores. An exception is the dimension 'relations and user rights' for which the regression analysis suggest a positive effect of a center being located in Barcelona.

We interpret the improvements as follows: first, the broad involvement of professionals in the design of consensus indicators and the participation of a large number of centers from the whole sector creates common sense and motivates improvement. Moreover, it allows setting challenging but realistic targets. Secondly, the combination of targeted feedback - to individual centers on the one hand and to the whole sector on the other - allows addressing confidentially those providers with low performance and, in collaboration with all stakeholders, bringing about change in the whole sector for those issues that require changes at the regulatory level. Both feedback mechanisms are consciously constructed, elaborated in collaboration with the participants and are supported by evidence-based guidance wherever possible. Moreover, center's feedback reports are addressed to the center's Chief Executive rather than individual professionals, and prepared in a way to support management in implementing change. Finally, the involvement of the whole sector and a wide range of stakeholders led to the development of small networks and collaborations between centers that promote the exchange of best-practice models and improvement projects.

However, a number of limitations of the study should be noted. First, we did not perform systematic psychometric validations of the measures used. Most of the indicators are based on existing measures previously validated and reported the literature. However, some measures also stem from the discussions and ratings by the consensus panels. While these measures have high face validity in the stakeholder group they may lack the presumed associations with outcomes, which should be addressed by future research [20]. Secondly, several factors might have confounded the improvements observed. As a starting point, this is the first evaluation of the sector that was carried out and it might be argued that centers started from a rather low baseline level. The improvements observed might thus reflect quality improvement actions that are easy to implement. Another factor explaining the improvements observed might be the three year time lag between baseline and follow up assessment. This time lag is on the one hand justified given the efforts to perform the assessment, provide center-specific and sector-wide performance feedback, implement quality improvement actions and prepare the follow up evaluation. On the other hand, this time lag also means that changes or evolutions in delivery, organization, or regulatory measures of social care might have influenced the results. Controlling for these potential confounding factors; however, would be in contradiction to our methodological approach which aims at involving all sector stakeholders and policy makers in addressing those quality problems that are beyond the control of an individual center. In this sense, our approach puts higher emphasis on working with the sector's stakeholders in addressing real life quality improvement actions (ecological validity) than on reducing threats to linking specific interventions to specific outcomes measures (internal validity) [21]. This is in line with what Berwick and quality improvement experts calls the 'pragmatic science' approach which gives importance to the context that shape the design and implementation of quality improvement strategies, rather than assuming that a 'best-model' exists that can be applied irrespective of contextual characteristics [22,23]. While maintaining the main orientations of our approach we plan for future evaluations to allow the applications of multi-level statistical models, which was not possible given the current algorithms for data collection [24]. Such an approach would allow a more in-depth analysis of the variations in improvements between and within centers, accounting for characteristics of users and organizational context.

## Conclusions

In conclusion, we report on an evaluation and improvement method for centers for attention and follow up of drugs users in Catalonia, Spain, in which importance is given to involve stakeholders in the development of performance measures and in which assessments are performed externally by independent evaluators. This method is designed to involve and motivate a whole sector in a given area. The results indicate that major improvements between baseline and follow up evaluation using these performance measure that are developed to 'make sense' for those being evaluated and that use standards that users themselves consider to be motivated and attainable. We believe that the broad involvement of stakeholders and the non-punishing nature of our approach to evaluation and improvement are key factors that might explain the substantial improvements observed. Further research should address how contextual issues shape the uptake and effectiveness of quality improvement actions, and how such quality improvements can be sustained.

## Competing interests

The authors declare that they have no competing interests.

## Authors' contributions

PH, RS, JC, RML and OG conceived of the study. OG performed the statistical analysis. OG, PH and RS drafted the manuscript. All authors critically read, revised and approved the final manuscript.
